# Investigating the light emitting diode (LED) flashlight characteristics of a sample of smartphones for its safety in indirect retinal photography

**DOI:** 10.11604/pamj.2022.43.15.32963

**Published:** 2022-09-08

**Authors:** Omar Mahmoud Solyman, Omnia Hamdy, Salwa Ahmed Abdelkawi, Aziza Ahmed Hassan

**Affiliations:** 1 Department of Ophthalmology, Research Institute of Ophthalmology, Giza, Egypt,; 2 Department of Ophthalmology, Qassim University Medical City, Qassim University, Al-Qassim, Saudi Arabia,; 3 Engineering Applications of Laser Department, National Institute of Laser Enhanced Sciences, Cairo University, Giza, Egypt,; 4 Biophysics and Laser Science Unit, Vision Science Department, Research Institute of Ophthalmology, Giza, Egypt,; 5 Medical Applications of Laser Department, National Institute of Laser Enhanced Sciences, Cairo University, Giza, Egypt

**Keywords:** Ophthalmic telemedicine, fundus photography, smartphone ophthalmoscopy

## Abstract

**Introduction:**

the off-label use of smartphones for indirect retinal photography and videography made it a popular ophthalmic clinical practice for its ubiquity and simplicity which enhanced telemedical care. Smartphone indirect retinal photography involves focusing the bright flashlight from the light emitting diode (LED) source on the rear side of the phone on the patient´s retina. Phototoxic hazards of the bright light on the already compromised patients´ retina raise concerns that require safety studies. The aim of this work is to study the characteristics of LED flashlights of a sample of smartphone types currently in use by ophthalmologists in Egypt to evaluate for potential photobiological implications when used in conjunction with + 20-diopter indirect ophthalmoscopy condensing lens for indirect photography of the retina.

**Methods:**

the spectral profile, weighted irradiance, and thermal exposure rates produced by a variety of smartphones´ LED flashlights currently used by ophthalmology specialists and trainees at the Comprehensive Outpatient Clinic of the Research Institute of Ophthalmology, Giza, Egypt, were tested in this study when collimated by a +20-diopter indirect ophthalmoscopy lens in a setup similar to actual indirect smartphone retinal photography.

**Results:**

the spectrum of the LED flashlights of all tested smartphones fell entirely in the optically safe visible spectrum between 400-750 nm with no significant infrared or ultraviolet components. Two regions of main spectral distribution were noticed in all tested smartphones with a peak at 450 nm and the other ranging between 520 to 585 nm. Weighted irradiance was within the safe limits for ocular examination and ranged from 0.58 to 2.30 mW/cm^2^ (safe limit is up to 706 mW/cm^2^) without a measurable thermal effect.

**Conclusion:**

the LED flashlight of the tested smartphones appeared to be within safe limits when used for indirect smartphone retinal photography. However, the high composition of the short wavelength blue light spectrum may be a concern particularly with prolonged and repeated examinations.

## Introduction

Ophthalmoscopic fundus examination is an important component of general medical examination and not only ophthalmic examinations. Ophthalmoscopy can reveal a myriad of ocular, systemic, and neurological diseases [1,2]. Smartphone ophthalmoscopy, which involves using of a smartphone´s rear-facing camera and built-in LED flashlight for indirect retinal and optic disc photography has become a popular practice thanks to its affordability, portability, and ease of use compared with conventional methods [3-7]. The built-in connectivity and post-processing capabilities, in addition to its ease to master made it delegable to paramedical staff/technicians and, hence, suitable for telemedicine. Integration of smartphone retinal photography with artificial intelligence/deep-learning algorithms will likely be the way forward in screening and diagnosing retinal and optic nerve disease such as diabetic retinopathy, age-related macular degeneration, retinopathy of prematurity, papilledema, etc. [8].

Smartphone retinal photography involves focusing the bright light of the LED flashlight source of the camera on the patient´s retina using an indirect ophthalmoscopy collimating lens. This raises concerns about the procedure´s safety on the patient´s retina which may be already compromised by the ongoing retinal pathology. The human eye is equipped with inherent protective mechanisms to guard against phototoxicity within certain limits. Overexposure to bright light can cause temporary or permanent damage to the retina and/or the retinal pigment epithelium (RPE) [9]. Light can induce damage to the neurosensory retina and the RPE through photothermal, photomechanical, and photochemical mechanisms. The risk of phototoxicity depends on the intensity, wavelength, and duration of light exposure and the mechanism can be multifactorial and overlapping [10]. The use of more powerful modern light sources in ophthalmic examination and surgery rendered photic injury a real danger, particularly in patients with compromised retina [11].

Irreversible thermal damage in the retina typically occurs after the ambient temperature in the retina is raised by at least 10°C, and the ability of light to induce such thermal damage is proportional to its wavelength [12]. Photomechanical damage results from the rapid introduction of energy into the melanosomes of RPE, resulting in shock waves that can result in permanent damage to the RPE or photoreceptors. The amount of damage is related to the delivery rate and the amount of energy absorbed [13]. Photochemical damage is thought to result from the exposure of retinal tissue to excessively generated reactive oxygen species (ROS). Photochemical damage is associated with long-duration exposure times as well as lower-wavelength light exposure and is the most common mechanism by which light exposure causes retinal damage [14].

There have been some concerns regarding the safety of the smartphone´s flashlight on the neurosensory retina and RPE [13,15]. A few older versions of smartphones´ flashlights had been tested for their safety for indirect retinal photography in a few prior reports, most recently in 2018 [13,15-17]. With the ongoing advancements in smartphone cameras and their associated brighter LED flashlights, studying the safety of newer generations of smartphones and previously untested smartphone versions are indicated [18]. In this study, we assess the safety of flashlights of various smartphones in current use by ophthalmology specialists and trainees at the Research Institute of Ophthalmology, Giza, Egypt, for indirect smartphone retinal photography.

## Methods

**Study setting:** it was conducted in the Biophysics Laboratory of the Research Institute of Ophthalmology, Giza, Egypt.

**Study design and period:** a facility-based physics laboratory study with no human or animal-based research conducted from March 1-31, 2021.

**Source population:** this study evaluated the physical characteristics of LED flashlight source of smartphones used by ophthalmologists for indirect smartphone retinal photography at the Research Institute of Ophthalmology Giza, Egypt for their safety when collimated on human retina.

### Eligibility criteria

**Inclusion criteria:** smartphones used for indirect retinal photography by ophthalmology specialists and trainees in the Comprehensive Outpatient Clinic at the Research Institute of Ophthalmology were borrowed to the biophysics laboratory to evaluate their LED flashlight characteristics.

**Exclusion criteria:** one representative smartphone of each smartphone version was tested to avoid duplicate data.

**Sample size determination and sampling technique:** the sample used here was based on the availability of the smartphones used by ophthalmology team at the Research Institute of Ophthalmology.

**Sampling procedures:** no sampling procedures were needed.

**Study variables, data collection tools and procedures:** the flashlight produced by each smartphone was collimated through a +20 diopter Volk double aspheric lens (Volk, Mentor, OH, USA) at a 25 cm distance from the phone. Then, condensed light was focused on an optical fiber with (SMA 905) interface to a digital fiber spectrometer (STDFSM, Touptek Photonics Co. Ltd, Zhejiang China) with a CCD (Toshiba TCD1304AP) and on GM 1040 portable digital Lux meter (Illuminator Mini Light, Hong Kong, Benetech). The resultant spectra were analyzed using the spectrometer operating software and Matlab R2018b. Light spectrum, intensity and thermal effect were tested for all studied smartphones at the highest available brightness with the battery fully charged and at medium level of brightness, when applicable.

**Data processing and analysis:** four versions of iPhones and five versions of Android-operated smartphones (Table 1) were evaluated in this study. Numerical data obtained from the spectrometer operating software and Matlab R2018b were represented in the form of bar and/or line graphs to be compared with the internationally recognised safe values to the eye.

**Table 1:** weighted irradiance of smartphones previously used for indirect retinal photography in the literature and those used in this study

Smartphone type	Measurements at maximum LED flashlight intensity (in mW/cm^2^)	Measurements at middle-level LED flashlight intensity when applicable (in mW/cm^2^)
iPhone 4	4.60 ^13^	
iPhone 6	1.40^15^; 1.38 *	0.65 *
iPhone 6+	1.40^15^; 1.30 *	0.75 *
Samsung note 7	1.91^16^	
Samsung S-4	1.28^16^	
Samsung S-5	1.27^16^	
Samsung grand	1.30^16^	
iPhone11	1.05 *	0.57 *
iPhone11 pro	1.09	0.60 *
Samsung note-8	2.30 *	1.26 *
Samsung A32	1.34 *	0.75 *
Samsung A12	1.09 *	
Hawai Y9	0.91 *	0.84 *
Oppo A54	0.58 *	
Oppo pro 6	0.94 *	

* this study; ** safe limit is one order of magnitude below maximum allowable level (706 mW/cm^2^)^19^

**Ethical approval and consent:** this is a non-human study. Ethical committee approval was not required for this type of studies.

## Results

**Figure 1:**
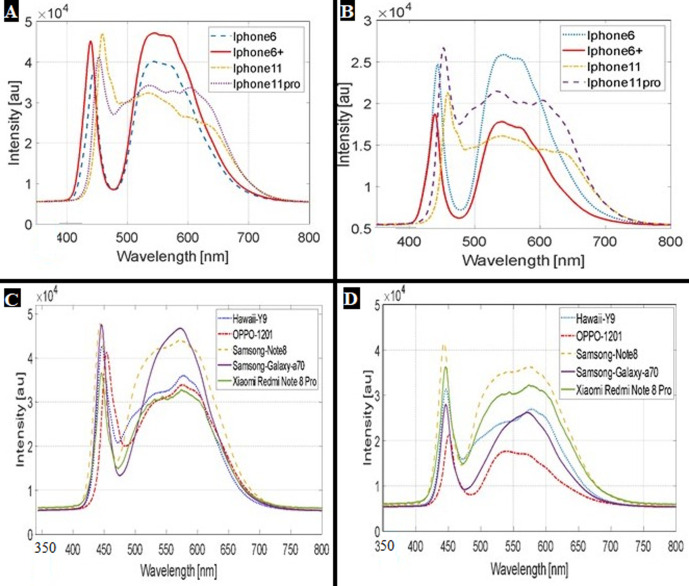
(A, B) the spectra of iPhones’ flashlights; (C, D) and Android smartphones tested in this study at their highest and medium intensity respectively

The spectrum of the LED light of all tested smartphones fell entirely in the optically safe visible spectrum between 400-750 nm with no significant infrared or ultraviolet components. Two regions of main spectral distribution were noticed in all tested smartphones with two peaks (Figure 1); the first around 450 nm and the second ranging between 520 to 585 nm. The irradiance of the produced blue light was within the safe limits for indirect retinal photography without a measurable thermal effect (Table 1).

## Discussion

The potential phototoxic effect of smartphone retinal photography has been studied on a limited number of phones in a few prior reports [13,15-17]. With the ongoing advancements in smartphone cameras and their associated brighter LED flashlights, studying of the safety of newer generations of smartphones and previously untested smartphone versions are indicated [18]. In this study, we evaluated the potential phototoxicity hazards of a variety of current versions of popular smartphones in current use by ophthalmology team members at the Research Institute of Ophthalmology for indirect retinal photography. Light spectrum, intensity and thermal effect were tested for all phones at the highest brightness and at middle-level brightness when applicable with the smartphone fully charged.

The light safety limits for ophthalmic instruments set by the International Organization for Standardization (ISO 15004-2.2) recommend that spectral irradiance on the retina be weighted separately for thermal and photochemical hazards. These safety limits are at least one order of magnitude below actual retinal threshold damage. The safety limit of retinal irradiance over a retinal area greater than 1.7 mm in diameter is 706 mW/cm^2^ [19]. Light intensity in this sample of smartphones was within safe limits for all smartphones tested in this study when used for indirect smartphone retinal photography at both middle-levels of brightness or the brightest flashlight intensity. In addition, prior reports showed weighted retinal irradiance of iPhone 4 [13], iPhone 6 and iPhone 6S [15] and a group of Samsung smartphones [16] to be within normal limits (Table 1).

The thermal effect of the LED of all tested smartphones in this study was non-measurable. This comes in accordance with prior studies and the fact that minimum thermal effect is one of the recognized advantages of LED light sources [20]. The spectrum of the LED light of all tested smartphones in this study fell entirely in the optically safe visible spectrum between 400-750 nm with no significant infrared or ultraviolet components. However, similar to prior studies [13,15-17], LED flashlights of tested smartphones showed high-level composition of the blue short-wavelength spectrum. Light-emitting diodes (LEDs) are superior to other light sources in terms of energy consumption, durability and temperature formation; however, the high blue shortwave length light output is a drawback [20].

Short-wavelength light has long been a concern for its potential photobiologic impact on the retina and the retinal pigment epithelial cells and has been postulated as a risk factor for the development of age-related macular degeneration (AMD). Short-wavelength light induces photochemical changes in the retina and the RPE which results in the production of ROS [20]. The resulting lifetime cumulative damage from ROS may be a contributing factor for AMD [21,22]. This raises a concern with prolonged and repeated examinations and photography given the known cumulative and additive effect of blue light hazards [23,24]. Several studies investigated the association between AMD progression and cataract surgery [25-28]. This association was hypothesized based on the fact that the short wavelength blockade by the yellow aging crystalline lens would be lost after cataract extraction. Hence yellow-tinged intraocular lenses have been introduced and widely used to reduce the presumable risk of AMD progression by blocking short wavelength blue light after cataract surgery [29]. Similarly, yellow-tinged indirect ophthalmoscopy lenses were introduced to decrease the load of short wavelength blue light focused on the retina during the ophthalmoscopy examination and to decrease patient discomfort [23]. Therefore, it is conceivable that the use of yellow-tinged indirect ophthalmoscopy lenses in conjunction with smartphone indirect retinal photography may be helpful in terms of reducing the blue shortwave light focused on the retina. A follow-up study for the effect of using yellow-tinged indirect ophthalmoscopy lens on the spectrum of LED smartphone light is recommended for potential protective effects and its effect on image quality.

**Limitations of this study:** these include the evaluation of a limited number of smartphones. Results may not be applicable to other untested smartphones. Another limitation of this study is the use of one type of non-tinted lens. While it is conceivable that the use of yellow-tinged indirect ophthalmoscopy lenses in conjunction with smartphone indirect retinal photography may be helpful in terms of reducing the blue shortwave light focused on the retina. A follow-up study for the effect of using yellow-tinged indirect ophthalmoscopy lens on the spectrum of LED smartphone light is recommended for potential protective effects and its effect on image quality.

## Conclusion

The LED flashlights of all tested smartphones in this study were found to be within the safe limits when used for indirect smartphone retinal photography. However, the high-level composition of the short-wavelength blue light spectrum may be concerning with prolonged and repeated examinations.

### What is known about this topic

- Smartphone indirect retinal photography has become a popular method of clinical documentation of retinal and optic disc findings;
- This method enhanced telemedicine thanks to its ease to master which made it delegable to paramedical staff/technicians, however, the safety of the bright smartphone´s LED flasshlight on the retina remains a concern;
- A few versions of smartphones´ flashlights were found to be safe when used for indirect retinal photography in prior reports most recently in 2018; a study of the safety of newer versions of smartphones´ flashlights may be indicated.

### What this study adds

- A wider range of currently used smartphones flashlights are within safe limits when used for indirect retinal photography;
- The high-level composition of short wavelength blue light component may be a concern with prolonged and repeated examination;
- This work suggests the use of once popular yellow-tinged indirect ophthalmoscopy condensing lenses to neutralize the high-level component of blue short wavelength light spectrum of the smartphones´ flashlights.
